# A Systematic Review and Meta-Analysis on Post-Abortion Contraceptive Utilization and Associated Factors in Ethiopia

**DOI:** 10.3389/fpubh.2022.883710

**Published:** 2022-05-20

**Authors:** Getu Engida Wake, Girma Wogie Fitie, Michael Amera Tizazu

**Affiliations:** Department of Midwifery, Institute of Medicine and Health Science, Debre Berhan University, Debre Berhan, Ethiopia

**Keywords:** pooled prevalence, prevalence, family planning, contraceptive, post-abortion, meta-analysis, Ethiopia

## Abstract

**Background:**

Post-abortion contraceptive utilization is the initiation and use of family planning methods at the time of management of abortion or before fertility returns. A significant discrepancy was reported regarding the prevalence and its associated factors of post-abortion contraceptive utilization in Ethiopia. So, this systematic review and meta-analysis aimed to estimate the pooled prevalence of post-abortion contraceptive utilization and its associated factors in Ethiopia.

**Methods:**

Preferred Reporting Items for Systematic Reviews and Meta-Analysis (PRISMA) guideline was used. The databases such as PubMed, Google Scholar, Science Direct, Cochrane library, Scopus, CINAHL, Web of Science, and additional searches by using direct Google search, libraries, and preprint were searched. Joanna Briggs Institute Meta-Analysis of Statistics Assessment and Review Instrument (JBI-MAStARI) was used for critical appraisal.

**Results:**

A total of 17 studies with 13,075 individuals were included. Of these, 14 studies with 5,719 individuals were used to estimate the prevalence. The pooled prevalence of post-abortion contraceptive utilization in Ethiopia was 63.64% (95% CI: 57.75–69.53). The subgroup analysis reported the highest prevalence of post-abortion contraceptive utilization in a study conducted in Addis Ababa (77.40%), a study published in 2015–2021 (66.15%), and among studies with a sample size >400 (66.84%). The pooled odds ratio (OR) of post-abortion contraceptive utilization for the mothers who had post-abortion family planning counseling was 4.15 (95% CI = 1.30, 13.2), and history of family planning utilization was 4.28 (95% CI = 2.66, 6.89).

**Conclusions:**

The pooled prevalence of utilization of post-abortion contraceptives in this meta-analysis remains low. Post-abortion family planning counseling and the history of the utilization of modern family planning methods were significantly associated with the practice of post-abortion contraceptives. The Ministry of Health should encourage post-abortion family planning utilization, making more efforts on post-abortion contraceptive counseling. Health facilities should work hard to strengthen the family planning counseling service, especially focusing on those who had no previous family planning utilization.

## Introduction

Abortion is the spontaneous or induced termination of pregnancy before fetal viability and the terms miscarriage and abortion are used interchangeably in a medical context (1). The post-abortion period is a time when women are highly interested to prevent or delay another pregnancy (2, 3). Post-abortion family planning (PAFP) is the initiation and use of family planning methods immediately after and within 48 h of abortion before fertility returns (4, 5) and it is a key component of post-abortion care (PAC) which includes voluntary contraceptive counseling and service provision (6).

Globally, maternal mortality is very high and nearly 2,95,000 women died from complications related to pregnancy and childbirth, and most of these deaths (94%) happened in low-resource settings (7). Sub-Saharan Africa (SSA) and Southern Asia accounted for ~86% of the estimated global maternal deaths (8). The World Health Organization (WHO) reported that ~8,00,000 women died each year worldwide because of abortion-related complications and this accounts for 19.6% of all maternal deaths (9, 10). Out of 35 million abortions that had occurred yearly in developing countries, 20 million were unsafe abortions and they account for the death of 67,000 women (11). Ethiopia has one of the highest maternal mortality ratios and low utilization of maternal health services in the world (12). Evidence indicated that unsafe abortion was the second most common cause of maternal mortality in Ethiopia, and it accounts for 19.7% of maternal deaths (13).

According to the World Health Organization (WHO) recommendation, a woman can safely use a full range of modern contraceptive methods immediately in the post-abortion period (14). Almost every death and disability related to abortion can be prevented through sex education, utilization of effective contraception, and the provision of legal abortion (15). Offering voluntary post-abortion family planning options generally minimize subsequent unplanned pregnancies and decrease maternal morbidity and mortality (9, 16). The lack of effective family planning counseling and services in the post-abortion period quickly leads to another induced abortion since fertility returns within 2–3 weeks after a miscarriage or induced abortion and this emphasizes the provision of quality post-abortion family planning counseling and service to all women presented for post-abortion care (17). All women within a reproductive age group should be provided quality family planning services including post-abortion contraceptive counseling immediately after abortion procedures equitably regardless of their age, marital status, and ethnicity (18), and the post-abortion period remains a golden and sensitive time to provide better understanding for the couples about family planning methods (19).

Different studies have been conducted in Ethiopia to determine the prevalence of post-abortion contraceptive utilization and its associated factors across the regions of the country beginning from August 2008 to mid-February 2021. But the results of those fragmented studies reported that there was great variability in the prevalence of utilization of post-abortion contraceptives (20–36). Concerning the associated factors, these studies indicated different maternal and health service-related factors such as post-abortion family planning counseling (20, 21, 23, 24, 26, 32, 36), maternal marriage (20, 22, 24–28, 34), previous contraceptive use (20, 22, 27), *age of mother* (22, 25–27, 29, 30), *history of abortion* (22, 26, 30), and partner support (35) as some of the determinant factors of utilization of post-abortion contraceptive. We selected post-abortion family planning counseling, maternal marital status, and previous history of contraceptive utilization from the above factors to investigate their effect on the practice of post-abortion contraceptives because of the following reasons:

The first reason is that offering high-quality and compassionate family planning counseling is very crucial to enabling couples to use modern contraceptives and meet their family planning needs and goals (37–39). Those mothers who had a history of contraceptive utilization had adequate awareness regarding some of the rumors about either experienced, anticipated, or rare complications which were falsely considered factual and affecting women's decision either not to choose, not to start, or discontinue contraceptives (40). The second reason is that the primary studies conducted previously showed controversial findings for all three selected outcomes. Among those original studies, some of them showed a negative association of maternal marriage with the practice of post-abortion contraceptives (22, 24, 26, 34), and the remaining showed a positive association of maternal marriage with the practice of post-abortion contraceptives with the presence of great variation among them (20, 25, 27, 28). Similarly, post-abortion contraceptive concealing (20, 21, 23, 24, 26, 32, 36) showed a positive and negative association with the practice of post-abortion contraceptives, respectively. Besides (20, 22, 27) showed a positive association of previous contraceptive utilization with the practice of post-abortion contraceptives with some variability among studies. Because of the aforementioned reasons, we intended to undertake this systematic review and meta-analysis. As far as our knowledge is concerned, there is no published systematic review and meta-analysis which investigated the pooled prevalence of post-abortion contraceptive utilization and its associated factors in Ethiopia using original studies published between August 2008 and mid-February 2021. So, the purpose of this systematic review and meta-analysis was to estimate the pooled prevalence of post-abortion contraceptive utilization and its association with post-abortion contraceptive counseling, maternal marriage, and previous contraceptive utilization in the context of Ethiopia. This systematic review will generate concrete evidence that helps policymakers and program planners to make an appropriate intervention and remold some policies concerning post-abortion contraceptive utilization and family planning counseling for the best benefits of mothers in Ethiopia.

## Methods

The preferred reporting items for systematic reviews and meta-analysis (PRISMA) guideline was used to evaluate the pooled prevalence of post-abortion contraceptive utilization and its association with post-abortion family planning counseling, maternal marriage, and previous contraceptive utilization in the context of Ethiopia in this systematic review and meta-analysis (41).

### Research Question/Hypothesis According to CoCoPop (Condition, Context, Population) Criteria

1. What is the pooled prevalence of post-abortion family planning utilization (PAFP) among mothers receiving abortion care services in the context of Ethiopia?
2. What is the association of post-abortion family planning utilization with post-abortion contraceptive counseling, maternal marriage, and previous family planning utilization among mothers receiving abortion care services in the context of Ethiopia?

### Searching Strategies and Databases

International databases such as the PubMed, Google Scholar, Science Direct, Cochrane library, Scopus, CINAHL, Web of Science, and additional searches by using direct Google search, libraries, and preprint were used. The search was conducted using the following search terms: (Utilization and determinants of post-abortion contraceptives in Ethiopia) OR (utilization OR magnitude OR prevalence) AND (determinants OR associated factors) AND (post-abortion) AND (contraceptive OR family planning) AND (Post-abortion family planning concealing OR post-abortion contraceptive counseling) AND (maternal marriage) AND (previous contraceptive utilization OR previous family planning utilization) AND Ethiopia. We conducted this search from 1 October 2021 to mid-February 2021, and all original studies published from August 2008 to mid-February 2021 were included.

### Eligibility Criteria

#### Inclusion Criteria

✓ Only studies conducted in Ethiopia
✓ All original articles published in peer-reviewed journals and stated in the English language
✓ Any observational study design which reported necessary information on the prevalence of post-abortion contraceptive utilization or those primary studies which reported data on the effect of family planning concealing, maternal marriage, or previous contraceptive utilization with post-abortion contraceptive utilization were included
✓ Only studies published from August 2008 to mid-February 2021 were considered.

#### Exclusion Criteria

✓ Studies with abstracts without full text, qualitative studies, and case reports
✓ Primary studies, which were not obtained even if the investigators made repetitive email contact, and all primary studies stated in a language other than English
✓ Experimental, intervention, and review articles

### Outcomes Measurement

This systematic review had four main outcomes. The primary outcome of this review was the pooled prevalence of post-abortion contraceptive utilization. The prevalence was calculated from each primary study by dividing the number of women who used post-abortion contraceptives by the total number of women who came for abortion service and multiplied by 100. The second, third, and fourth outcomes were to examine the association of family planning counseling, maternal marital status, and contraceptive utilization history with the primary outcome, respectively. We calculated the Ln odds ratio (Ln OR) based on the results of the original studies that examined the relationship between the above three variables with the primary outcome.

### Data Extraction

Three independent reviewers (GW, GF, and MT) extracted the data and cross-checked them to ensure consistency using a standardized data extraction format which was adopted from the JBI data extraction format. Any unclear information and disagreement which arose between the reviewers during data abstraction were resolved through discussion and agreement. Data were collected on the following variables: primary author, publication year, study area, study design, sample size, the prevalence with 95% CI, and the quality score of each primary study.

### Quality Assessment

The quality of each original article was evaluated using Joanna Briggs Institute critical appraisal tools for use in JBI systematic reviews (JBI-MAStARI) of cross-sectional studies (42), which have 8 major criteria. Accordingly, primary studies with a score of ≥50% and above were included in the meta-analysis research.

### Statistical Analysis

Data were extracted in the Microsoft Excel format and analysis was done using STATA version 11 software. We calculated the standard error for each original study using the binomial distribution format. Heterogeneity regarding reported prevalence was assessed by computing the *p*-values for Cochrane Q-statistics and *I*^2^ tests. The *I*^2^ test statistics of 25, 50, and 75% were declared of low, moderate, and high heterogeneity, respectively (43). A random-effect meta-analysis model was used to estimate the overall prevalence of post-abortion contraceptive utilization. To minimize the random variations between primary studies, subgroup analysis was done by regions in Ethiopia, sample size, and publication year of primary studies. Besides, univariate meta-regression was conducted considering the year of publication of original studies, the total sample size, and the region in Ethiopia as covariates to identify the possible sources of heterogeneity, and sensitivity analysis was employed to examine the effect of a single study on the overall estimation. We checked publication bias using a funnel plot subjectively and Begg's and Egger's tests objectively and a *p*-value of < 0.05 was used to declare the statistical significance of publication bias (44). A point prevalence and its 95% confidence intervals were presented with the forest plot. Accordingly, the size of each box corresponded to the weight of the study, the crossed line referred to a 95% confidence interval of the study, and the Ln odds ratio (Ln OR) was applied to examine the association of post-abortion contraceptive counseling, maternal marriage, and previous utilization of contraceptive with post-abortion contraceptive utilization in Ethiopia.

## Results

### Search Results

**Figure 1:**
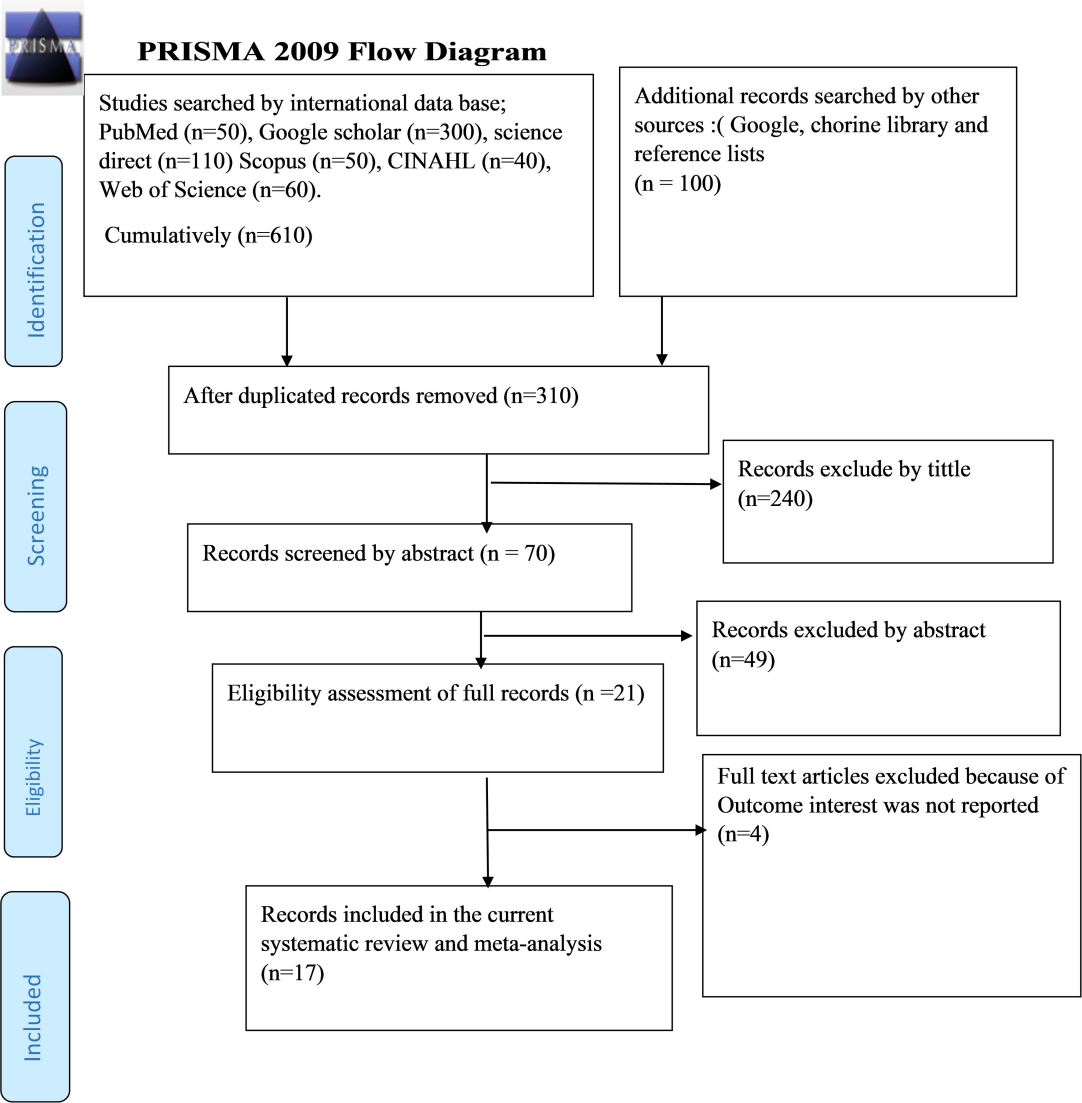
Flow diagram of studies included in systematic review and meta-analysis.

As described in Figure 1, 710 studies were identified regarding the prevalence of post-abortion contraceptive utilization and its associated factors in Ethiopia through PubMed, Google Scholar, Science Direct, Scopus, CINAHL, Web of Science, and additional searches by using direct Google search, libraries, and preprint. Then 400 studies were excluded because of duplication. From the remaining 310 studies, 240 articles were excluded after reviewing their titles based on assessment because of lack of relevance to the current study. The rest 70 records were screened by abstracts and this made 49 records to be excluded. Furthermore, 21 full-text records were screened for the fulfillment of the stated inclusion criteria. After all, four articles were excluded due to lack of fulfillment of inclusion criteria. Amid excluded studies, all four studies were excluded because of the missing outcome of interest. Eventually, 17 studies were included in the systematic review and meta-analysis.

### Study Characteristics

As displayed in Table 1, the entire of these 17 original studies were published between August 2008 and mid-February, 2021. A total of 13,075 post-abortion mothers were included in our meta-analysis and systematic review. All of those studies were cross-sectional study designs. The lowest and highest sample sizes of the included studies were 103 and 5,604, respectively. A study conducted in the Oromia and Amhara region (44.66%) (31), and a study conducted in Addis Ababa, Ethiopia (90.5%) (28), reported the lowest and highest prevalence of post-abortion contraceptive utilization, respectively. Primary studies from five administrative regions and one council city were included in this meta-analysis from nine administrative areas (regions) of Ethiopia. Among the included primary studies, six were from Amhara (22–25, 32, 36), two from Tigray (20, 21), four from Addis Ababa (28–30, 37), one from Oromia (27), one from Gambella (26), one from Oromia and Amhara region in combination (31), one from Southern nations, nationalities, and people (SNNP) (33), and one from Nationwide (34). No studies were reported from Benishangul Gumz, Harari, Somalia, Afar, and Dire Dawa council city. Regarding the quality score of each primary study, the score was between the lowest five and highest eight and none of the studies were excluded based on the quality assessment criteria (Supplementary File 1) and almost all primary studies had a sufficient response rate.

**Table 1:** Descriptive summary of 17 studies included in the meta-analysis of the prevalence of post-abortion contraceptive utilization in Ethiopia.

**S.no**	**References**	**Study area**	**Study design**	**Sample size**	**Prevalence PAFP**	**Response rate (%)**	**Study participant characteristics**	**Family planning counseling (OR)**	**Maternal marital status (OR)**	**Previous utilization of contraceptives (OR)**
1	Moges et al. (20)	Tigray	Cross-sectional	400	61.5	98	Among women receiving abortion service	3.53	2.59	3.62
2	Hagos et al. (21)	Tigray	Cross-sectional	409	70.9	98.3	Among women receiving abortion services	1.8	–	–
3	Mekuria et al. (22)	Amhara	Cross-sectional	400	78.5	96.4	Among women after abortion	–	0.14	4.73
4	Muche et al. (23)	Amhara	Cross-sectional	371	45.8	100	Women who came for abortion service	19.245	–	–
5	Kokeb et al. (24)	Amhara	Cross-sectional	414	59.2	100	Among women came for abortion service	4.2	0.56	–
6	Seid et al. (25)	Amhara	Cross-sectional	282	47.5	97.0	Post-abortion women	–	1.14	–
7	Abamecha et al. (26)	Gambella	Cross-sectional	399	72.9	97.8	Among women came for abortion service	0.303	0.45	–
8	Erko et al. (27)	Oromia	Cross-sectional	184	70.1	90.2	Women seeking abortion care services	–	6.711	6.4
9	Asrat et al. (28)	Addis Ababa	Cross-sectional	552	90.5	97.4	Women who received post-abortion care service	–	3.31	–
10	Prata et al. (29)	Addis Ababa	Cross-sectional	1,200	77.6	100	Women seeking abortion-related services	–	–	–
11	Prata et al. (30)	Addis Ababa	Cross-sectional	1,200	86	100	Women seeking abortion-related services	–	–	–
12	Kumbi et al. (31)	Oromia and Amhara	Cross-sectional	103	44.66	100	Post-abortion clients	–	–	–
13	Abate et al. (32)	Amhara	Cross-sectional	423	64.77	100	Clients who came for either spontaneous or induced	25.47	–	–
14	Tesfaye et al. (33)	SNNP	Cross-sectional	400	56.5	95	Post-abortion clients	–	–	–
15	Wado et al. (34)	National	Cross-sectional	5,604	74.78	100	Post-abortion clients	–	0.78	–
16	Teshome et al. (35)	Addis Ababa	Cross-sectional	326	76.4	100	Among women receiving abortion care	–	–	–
17	Muchi et al. (36)	Amhara	Cross-sectional	408	61	100	Among women who seek abortion service	5.99	–	–

### Meta-Analysis

#### Publication Bias

**Figure 2:**
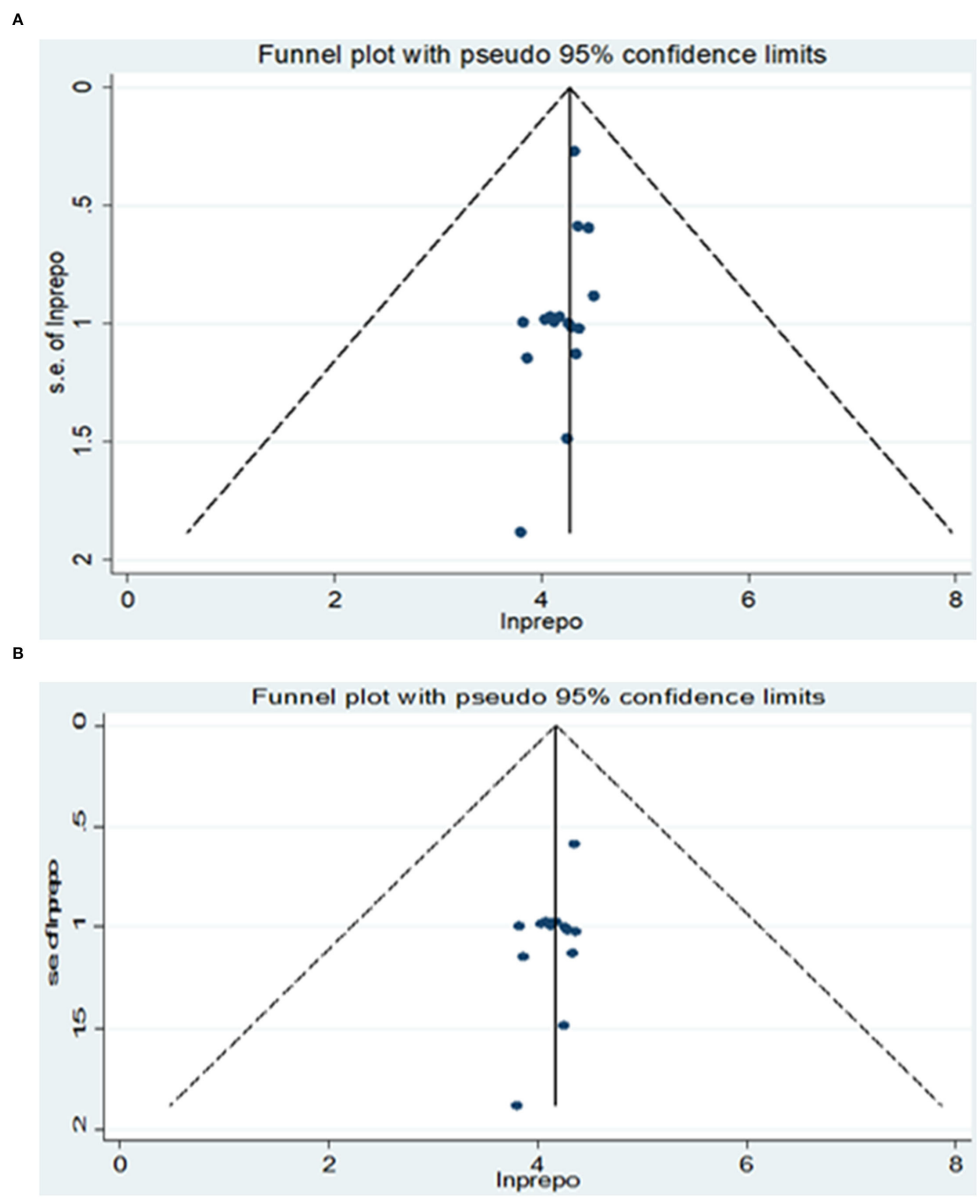
**(A)** Funnel plot for publication bias of 17 primary studies to estimate the pooled prevalence of PAFP utilization in Ethiopia. **(B)** Funnel plot for publication bias of 17 primary studies to estimate the pooled prevalence of PAFP utilization in Ethiopia.

From the total of 17 original studies, three studies (28, 30, 34) were excluded from prevalence estimation using leave-one-out sensitivity analysis to identify the potential source of heterogeneity in the analysis after checking the funnel plot asymmetry and the significance of Egger's regression tests. These studies were Asrat et al.'s (28), which reported the prevalence of post-abortion family planning (PAFP) 90.5% in Addis Ababa, Prata et al.'s (30), which reported the prevalence of post-abortion family planning (PAFP) 86% in Addis Ababa, and Wado et al.'s (34) reported 74.8% of prevalence of PAFP. However, they were not excluded from the meta-analysis for associated factors. Significant publication bias with an Egger's regression *p*-value < 0.039 was seen when all studies were considered (Figure 2A). After adjustment, Egger's regression *p*-value was 0.078, which indicated a reduced publication bias (Figure 2B).

### The Pooled Prevalence of Post-Abortion Contraceptive Utilization

**Figure 3:**
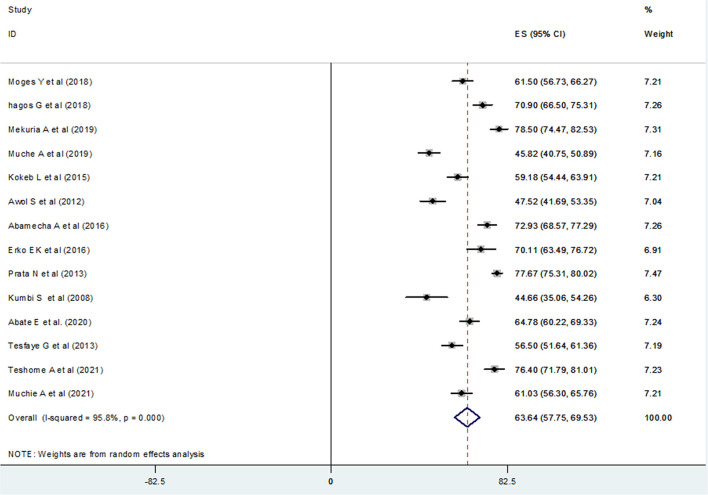
Forest plot of the pooled prevalence of post-abortion contraceptive utilization of 14 studies in Ethiopia.

Consequently, fourteen studies (20–27, 29, 31–33, 35, 36) (*n* = 5,719 mothers) were included in the final meta-analysis, giving the pooled prevalence of post-abortion contraceptive utilization in Ethiopia 63.64% (95% CI: 57.75–69.53) (Figure 3). Because of high heterogeneity among the included studies (*I*^2^ 95.8%, *p* = < 0.001) the random-effect meta-analysis model was used. In our meta-analysis research, Mekuria et al. (78.50%) and Kumbi et al. (44.66%) reported the highest and lowest prevalence of post-abortion contraceptive utilization, respectively. The Begg's and Egger's regression test *p*-value of this review was 0.822 and 0.078, respectively. Besides, we conducted a univariate meta-regression by considering the year of publication, total sample size, and region in Ethiopia as covariates to identify the possible sources of heterogeneity, but unfortunately, none of them were found to be statistically significant (Table 2).

**Table 2:** Heterogeneity of post-abortion contraceptive utilization in the current meta-analysis (based on univariate meta-regression considering the year of publication, sample size, and regions in Ethiopia as a covariate).

**Variables**	**Coefficient (individual)**	***p*-value (individual)**
Year of publication	0.0251	0.812
Sample size	0.00036	0.689
Regions in Ethiopia	0.005	0.935

### Subgroup Analysis

**Figure 4:**
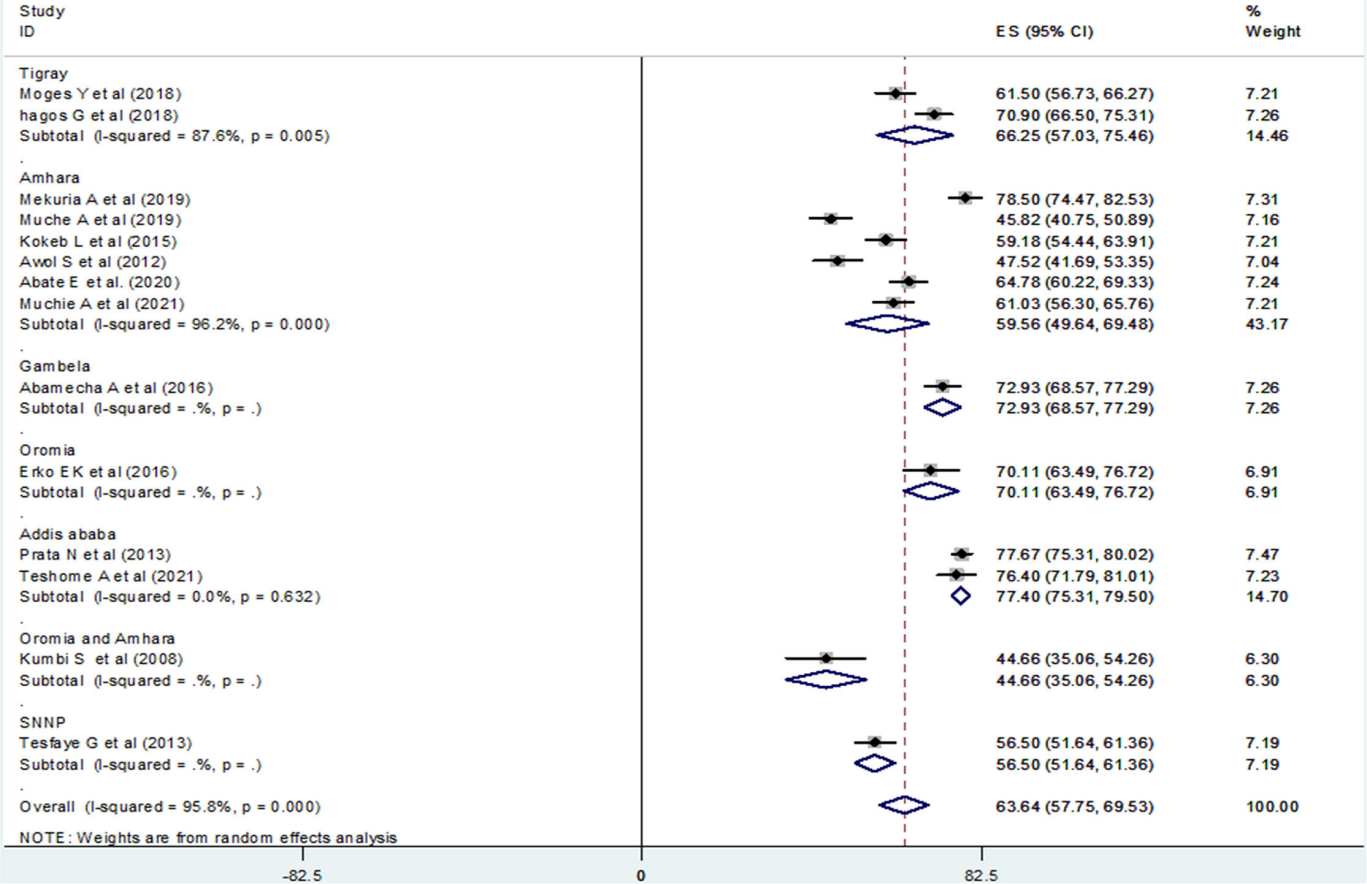
Forest plot of the prevalence of PAFP with corresponding 95% CIs of the subgroup analysis based on the regions in Ethiopia.

**Figure 5:**
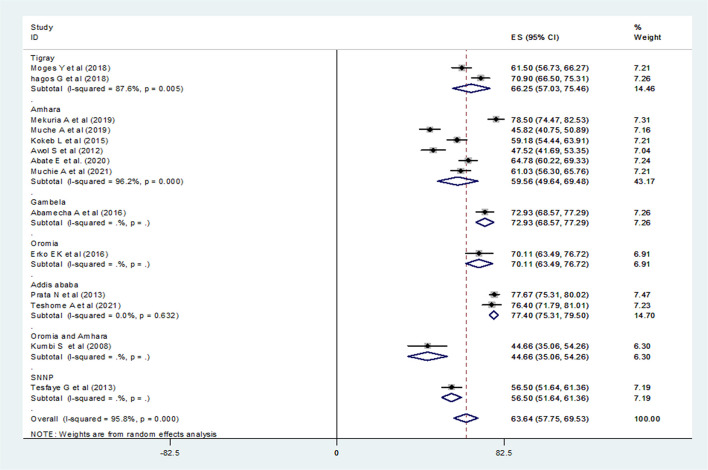
Forest plot of the prevalence of PAFP with corresponding 95% CIs of the subgroup analysis based on the year of publication in Ethiopia.

**Figure 6:**
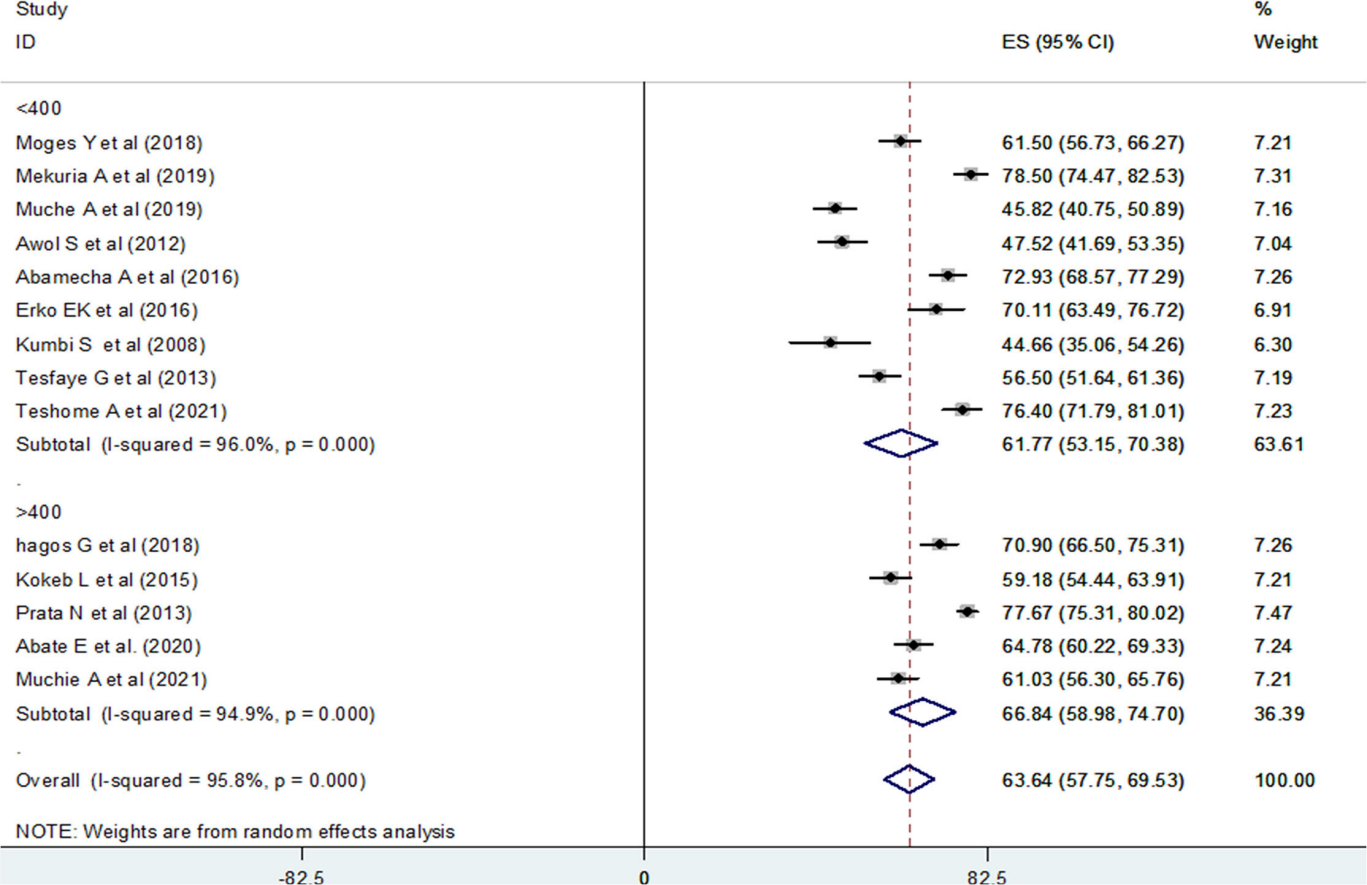
Forest plot of the prevalence of PAFP with corresponding 95% CIs of the subgroup analysis based on the total sample size of studies in Ethiopia.

A subgroup analysis was done by administrative regions in Ethiopia, the year of publication of original studies, and the total sample size of primary studies to compare the prevalence of post-abortion contraceptive utilization across different studies. Because of high heterogeneity, a random-effect model meta-analysis was done for all three variables. Accordingly, this systematic review and meta-analysis reported the highest prevalence of post-abortion contraceptive utilization in a study conducted in Addis Ababa at 77.40% (95% CI: 75.31, 79.50) (Figure 4), a study published during 2015–2021, 66.15% (95% CI: 60.12, 72.17) (Figure 5), and among studies with a sample size >400 (66.84%) (95% CI: 58.98, 74.70) (Figure 6).

### Association of Post-Abortion Family Planning Counseling With the Utilization of Post-Abortion Contraceptives in Ethiopia

**Figure 7:**
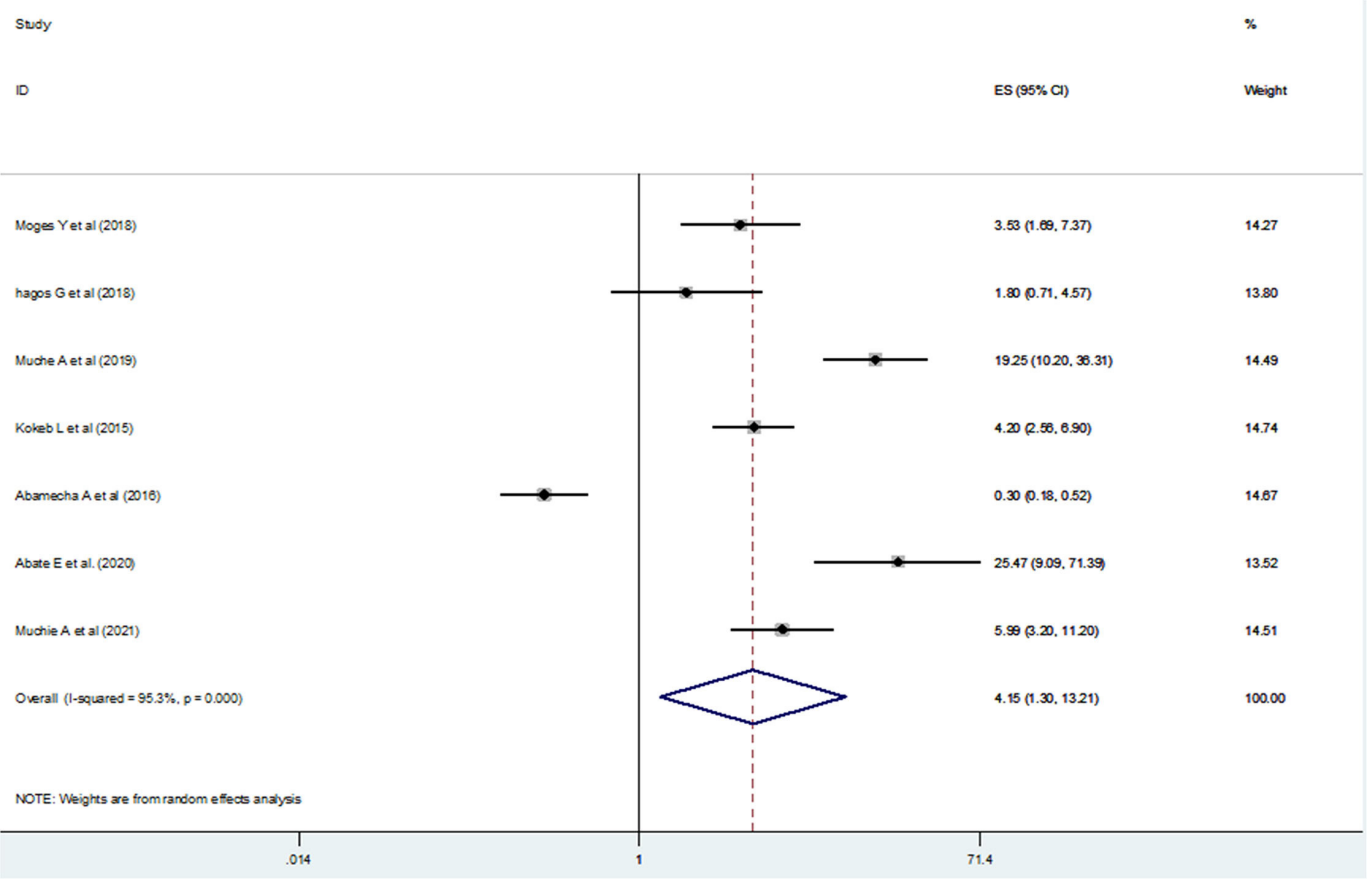
The pooled odds ratio of the association between post-abortion contraceptive counseling and post-abortion contraceptive utilization in Ethiopia, 2021.

**Figure 8:**
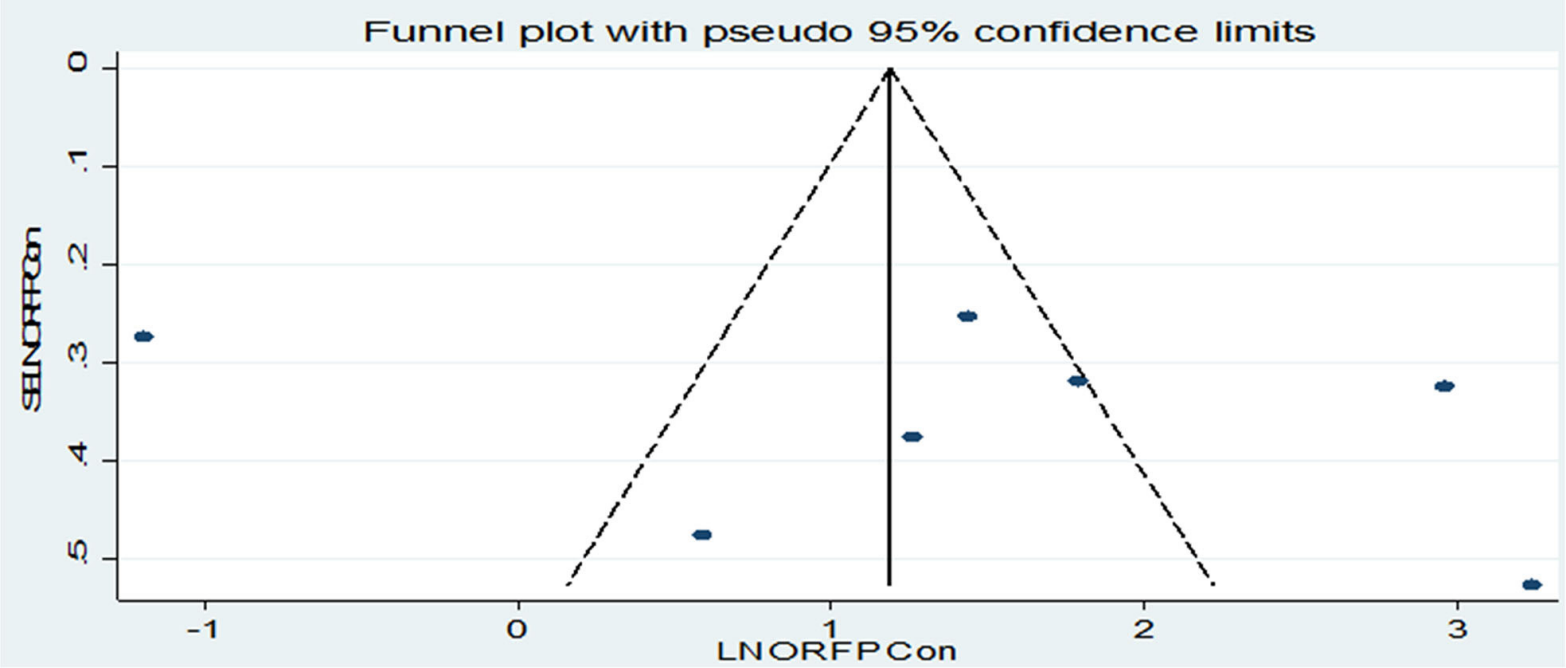
Funnel plot for publication bias, LNORFPCON represented in the *x*-axis and standard error of SELNORFPCON on the *y*-axis.

We examined the association between post-abortion contraceptive counseling and the practice of post-abortion contraceptives using seven studies (20, 21, 23, 24, 26, 32, 36). Since high heterogeneity (*I*^2^ = 95.3% and *p*-value < 0.001) was observed across the included studies, a random-effect meta-analysis model was applied and the finding revealed that the practice of post-abortion contraceptive utilization was significantly associated with maternal post-abortion contraceptive counseling [(OR) 4.15; 95% CI (1.30–13.2)] (Figure 7). We also assessed publication bias using the funnel plot and Begg's and Egger's tests. Even if the funnel plot showed the presence of publication bias (Figure 8), Begg's and Egger's tests showed the absence of a significant publication bias (*p*-value > 0.764 and *p* = 0.442), respectively.

### Association of Maternal Marital Status With the Utilization of Post-Abortion Contraceptive

**Figure 9:**
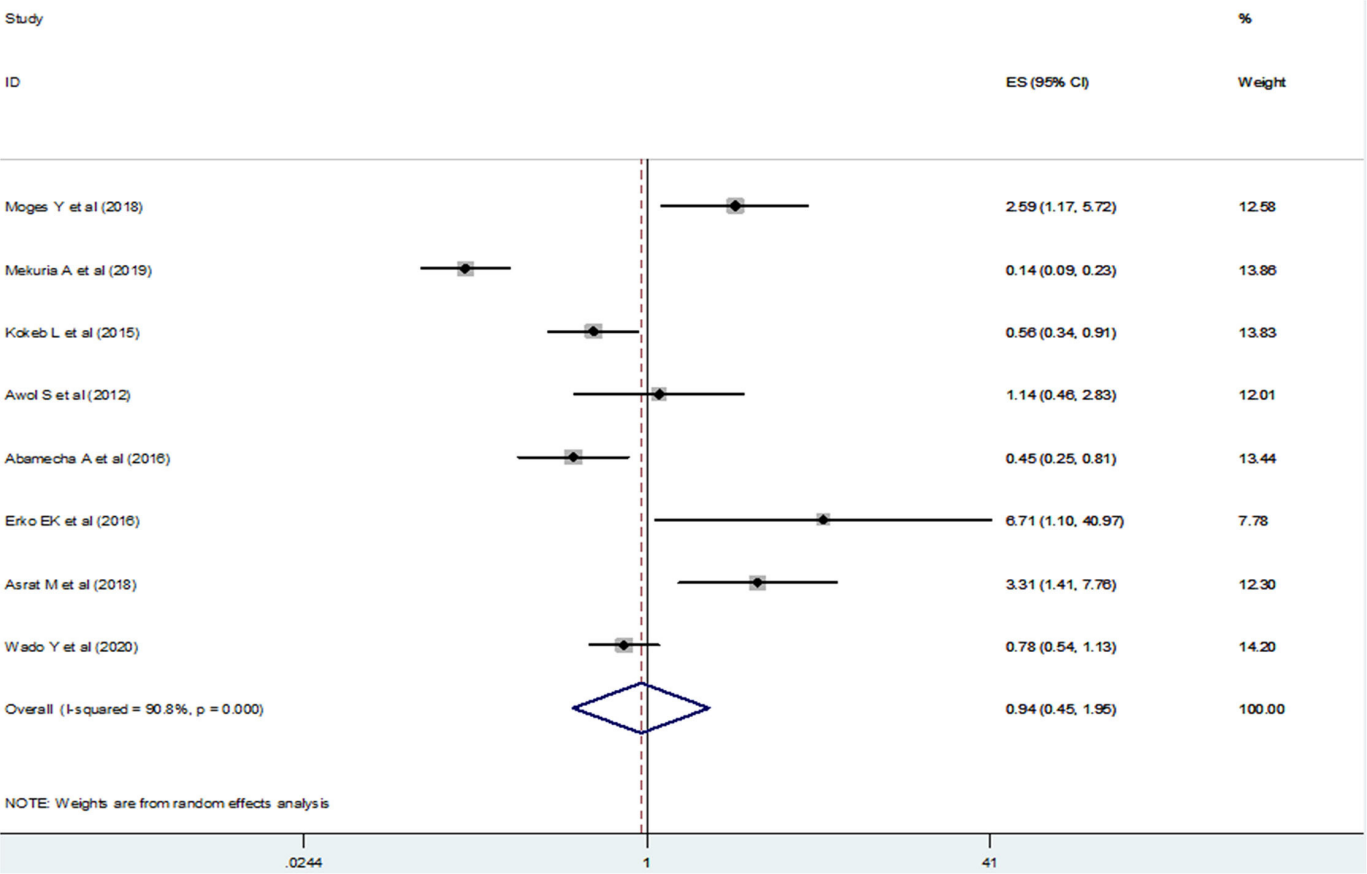
The pooled odds ratio of the association between maternal marital status and post-abortion contraceptive utilization in Ethiopia.

**Figure 10:**
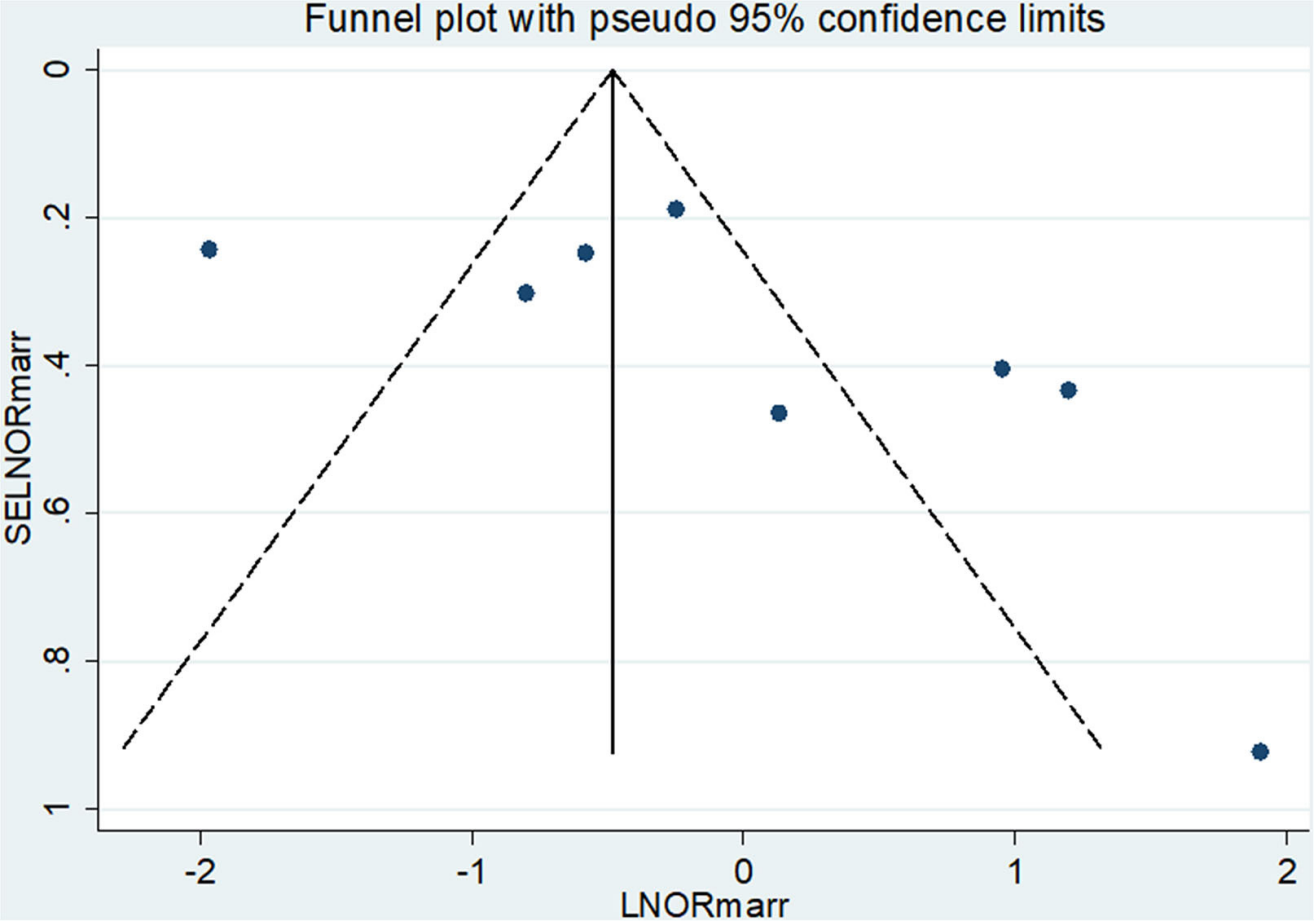
Funnel plot for publication bias, LNORmarr represented in the *x*-axis and standard error of SELNORmarr on the *y*-axis.

We examined the association between maternal marital status and the practice of post-abortion contraceptives using eight studies (20, 22, 24–28, 34). Since high heterogeneity (*I*^2^ =90.8% and *p*-value < 0.001) was observed across the included studies, a random-effect meta-analysis model was applied and the finding of those included studies showed that post-abortion contraceptive utilization was not significantly associated with maternal marriage status [(OR) 0.94; 95% CI (0.45–1.95)] (Figure 9). We assessed publication bias using Begg's and Egger's tests and even if the funnel plot showed the presence of publication bias (Figure 10), Begg's and Egger's tests showed the absence of a significant publication bias (*p*- value > 0.266 and *p* = 0.163), respectively.

### Association of Previous Family Planning Practice With the Utilization of Post-Abortion Contraceptive

**Figure 11:**
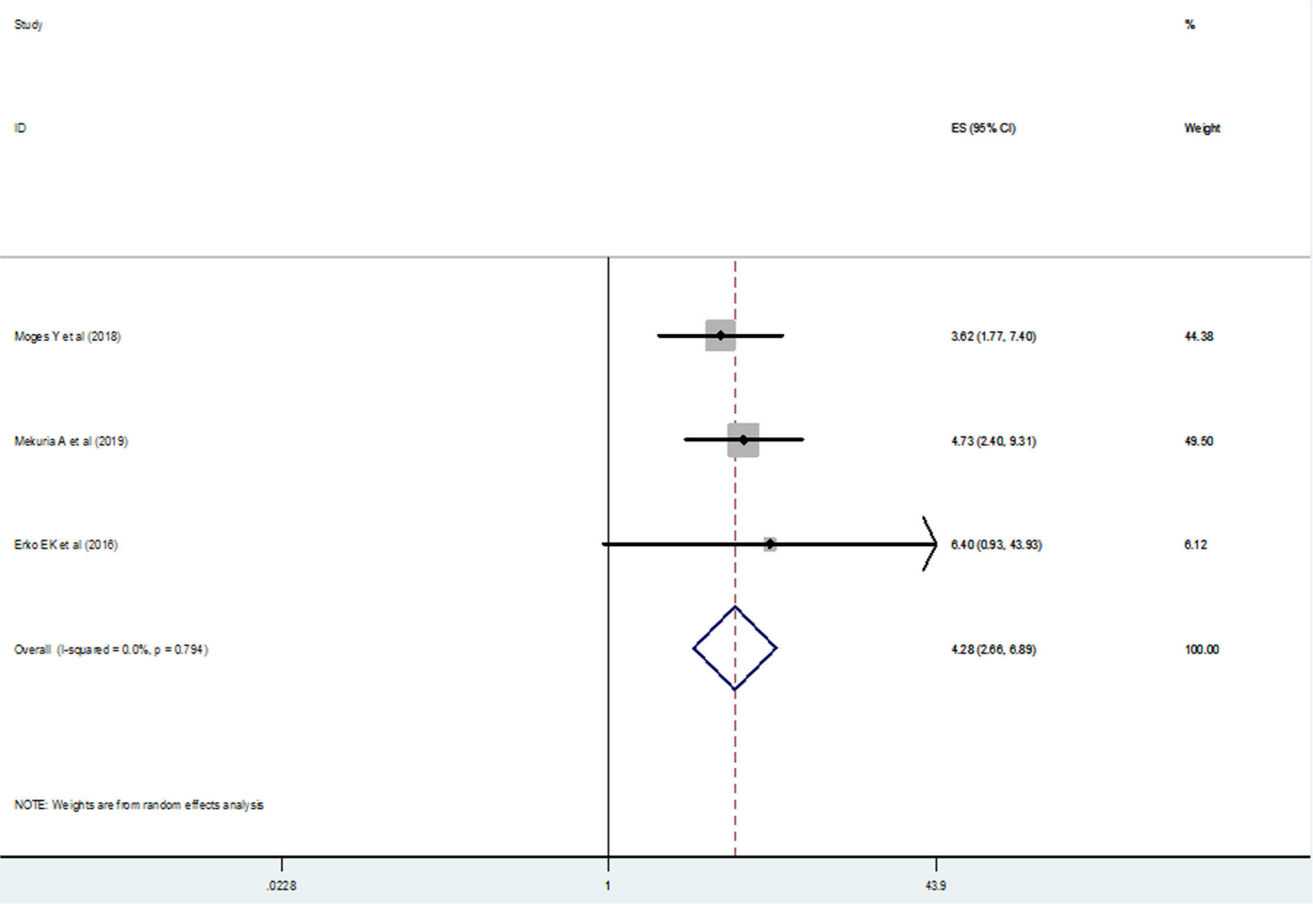
The pooled odds ratio of the association between previous utilization of family planning by mothers and post-abortion contraceptive utilization in Ethiopia.

We examined the association between the previous history of family planning utilization among mothers and post-abortion contraceptive utilization using 3 primary studies (20, 22, 27) and those mothers who had a previous history of contraceptive utilization were four times more likely to practice post-abortion contraceptives in comparison to their counterparts [(OR) 4.28; 95% CI (2.66–6.89)] (Figure 11). Similarly, publication bias was assessed using funnel plot and Begg's and Egger's tests. Unfortunately, Begg's and Egger's tests showed the absence of a significant publication bias (*p*-value > 1.00 and *p* = 0.612), respectively.

## Discussion

To prevent any complications arising from pregnancy and childbirth and to safeguard their health, women have the right to get family planning (FP) education and information (45). Family planning and other reproductive health services play a pivotal role to meet achievements toward securing safe drinking water, sanitation, adequate housing, and the other sustainable development goals (SDGs) components (46). This systematic review and meta-analysis estimated the national pooled prevalence of post-abortion contraceptive utilization in Ethiopia. Accordingly, the pooled prevalence of utilization of post-abortion contraceptives in Ethiopia was 63.64%(95% CI: 57.75–69.53) and this result was lower than the finding of the study conducted in Kenya 76% (47), two studies from Tanzania 86% (48) and 90% (49), Somalia 88% (50), Guinea 91.1% (51), Sub-Saharan countries 73% (52), Asia and sub-Saharan countries 77% (53), Pakistan 72.9% (54), Brazil 97.4% (55), Turkey 75.9% (9), and India 81% (56). This difference might be due to methodological differences such as data analysis and sampling of study participants, variation in maternal socio-demographic characteristics, and economic and health service utilization differences. But, the pooled prevalence of post-abortion contraceptive utilization in this meta-analysis was higher than the result of the study conducted in Nepal 49.5% (57). This difference might be related to the implementation of different health sector development programs (HSDP) in Ethiopia (58) and heavy investment in the healthcare system which resulted in significant gains in the improvement of the health status in Ethiopia (59).

Besides, we performed a subgroup analysis based on the study areas (regions) in Ethiopia where the studies were conducted. Accordingly, the highest (77.40%) and lowest (44.66%) prevalence of post-abortion contraceptive utilization was reported among studies conducted in Addis Ababa and Oromia and Amhara (in combination), respectively. This difference might be attributed to the number of studies included in each category during analysis, the time gap at which those original studies were conducted, and the difference in coverage of health information regarding post-abortion contraceptive utilization. Again, this difference could be due to maternal residence, and in our review, the study participants of the studies conducted in Addis Ababa scored higher prevalence of utilization of post-abortion contraceptives which indicated the effect of urbanization. Urbanization is one of the major factors associated with effective health care services utilization compared to rural residents and some works of the literature showed that there was a variation in the utilization of health care services among rural and urban dwellers (60, 61). We also performed a subgroup analysis based on the publication year of the original studies. Accordingly, the highest (66.15%) and lowest (56.85%) prevalence of post-abortion contraceptive utilization was reported among studies conducted in 2015–2021 and in 2008–2014, respectively. This might be attributed to the Ethiopian government's commitment and hard work to meet the sustainable development goals and revision of the 2016–2020 national reproductive health strategy (RHS) which stated the goal of family planning is to reduce unintended pregnancies and enable individuals to achieve their desired family size (62). Another possible explanation was that during 2015–2019, the Ethiopian government set a health sector transformation plan (HSTP) which was in line with the goals of SDG, intensifying reproductive, maternal, newborn, child, and adolescent health (RMNCAH) interventions to end preventable maternal and child deaths by 2030 (59).

In addition, we conducted a subgroup analysis based on the total sample size of the primary studies. The finding showed that the highest (66.84%) and lowest (61.77%) prevalence of post-abortion contraceptive utilization was reported among primary studies with a sample size of >400 and <400, respectively. Evidence showed that a larger sample size provides greater reliability/precision in the estimates of the effect of the study (63). One of the greatest threats to the validity of meta-analytic results is publication bias, which generally leads to effect sizes being overestimated and the dissemination of false-positive results (64–66). Because of this, we assessed the possible sources of heterogeneity using univariate meta-regression, and unfortunately, the test showed no statistical significance.

One of the pillar components of post-abortion care (PAC) is family planning counseling and service provision (67). Lack of effective post-abortion family planning counseling and services delivery quickly leads to another induced abortion. Because of this, we intended to assess the effects of post-abortion contraceptive counseling on the utilization of post-abortion contraceptives among women who came for abortion services. Those mothers who had post-abortion family planning counseling were four times more likely to use post-abortion contraceptives. This finding was similar to the results of the study conducted in Ghana (68), Turkey (9), and two studies in Brazil (69, 70). This emphasizes that family planning counseling is one of the critical elements in the provision of quality family planning services, which enables women to make informed decisions and post-abortion contraceptive counseling is an effective way of increasing the utilization of post-abortion contraceptives (71, 72).

According to this study's finding, there was no difference in the utilization of post-abortion contraceptives among married and unmarried women. This might be because women are becoming more autonomous and exercising their reproductive rights. Evidence showed that today's married women are more autonomous and decide to use any type of contraceptive they prefer in Ethiopia (73). Most of the study participants in our review were from urban areas and women from urban areas are more autonomous in deciding their contraceptive choice independently (74). Those mothers who had a history of contraceptive utilization were more than four times more likely to use a post-abortion contraceptive. This finding was similar to the results of the studies conducted in Tanzania (75), Zambia (76), and Pakistan (54). This could be because women who had a history of contraceptive utilization had adequate awareness of some of the rumors and fears of side effects which were leading factors for women either not to choose, not to start, or discontinue contraceptive methods (77–79).

## Strengths and Limitations of This Study

This study is the first systematic review and meta-analysis research estimating the national pooled prevalence of PAFP in Ethiopia. The evidence from this systematic review and meta-analysis could be taken as input for health care policymakers, researchers, and health practitioners. Since studies from some regions were lacking, the results might not be representative of the entire country. This systematic review and meta-analysis was not registered in PROSPERO.

## Conclusions

The pooled prevalence of utilization of post-abortion contraceptives in Ethiopia remains low. Post-abortion family planning counseling and the history of utilization of modern family planning methods were significantly associated with the practice of post-abortion contraceptives. Based on our review findings, we strongly recommended that the Ministry of Health should encourage post-abortion family planning utilization, making more efforts on post-abortion contraceptive counseling for the sustainable practice of PAFP. The governmental and non-governmental organizations should work to upgrade post-abortion contraceptive utilization. Health facilities should work hard to strengthen the family planning counseling service, especially focusing on those who had no previous family planning utilization history in addition to other post-abortion care services.

## Data Availability Statement

The datasets presented in this study can be found in online repositories. The names of the repository/repositories and accession number(s) can be found in the article/Supplementary Material.

## Author Contributions

GW: conceptualization of the research protocol, study design, literature review, data extraction, data analysis, interpretation, and drafting of the manuscript. GF and MT: data extraction, quality assessment, data analysis, reviewing the manuscript, and rewriting review. All authors have read and approved the manuscript for publication.

## Conflict of Interest

The authors declare that the research was conducted in the absence of any commercial or financial relationships that could be construed as a potential conflict of interest.

## Publisher's Note

All claims expressed in this article are solely those of the authors and do not necessarily represent those of their affiliated organizations, or those of the publisher, the editors and the reviewers. Any product that may be evaluated in this article, or claim that may be made by its manufacturer, is not guaranteed or endorsed by the publisher.

## Abbreviations

AIDS: acquired immune deficiency syndrome
CI: confidence interval
DHST: demographic and health survey of Tanzania
EDHS: Ethiopian demographic health survey
FP: family planning
HICs: high-income countries
HIV: human immunodeficiency virus
HSDP: health sector development program
HSTP: health sector transformation plan
JBI: Joanna Briggs Institute
JBI-MAStARI: Joanna Briggs Institute Meta-Analysis of Statistics Assessment and Review Instrument
MMR: maternal mortality ratio
NRHS: national reproductive health strategy
OR: odds ratio
PAC: post-abortion care
PAFP: post-abortion family planning
PRISMA: preferred reporting items for systematic reviews and meta-analyses
PROSPERO: International prospective register of systematic reviews
RMNCAH: reproductive, maternal and newborn, child, adolescent health
SDG: sustainable development goal
SNNP: Southern nations, nationalities, and Peoples'
SSA: sub-Saharan African
UN: United Nation
WHO: World Health Organization.

## References

[1] Cunningham G Leveno KJ Bloom SL Spong CY Dashe JS . Williams Obstetrics. 24th ed. New York, NY: McGraw-Hill (2014). p. 350–70. Available online at: http://bit.ly/2KW2IFY (accessed April 6, 2022).

[2] FIGO, ICM, ICN, USAID, WRA, DFID . Post-Abortion Family Planning: A Key Component of Post Abortion Care. Consensus Statement: International Federation of Gynecology and Obstetrics (FIGO), International Confederation of Midwives (ICM), International Council of Nurses (ICN), United States Ag. (2013). p. 1–4. Available online at: http://www.respond-project.org/pages/files/6_pubs/advocacy-materials/PAC-FP-Joint-Statement-November2013-final.pdf (accessed April 6, 2022)

[3] Gizaw A Regassa N. Family planning service utilization in Mojo town, Ethiopia: a population-based study. J Geogr Reg Plan. (2011) 4:355–63. (accessed April 6, 2022). Available online at: https://citeseerx.ist.psu.edu/viewdoc/download?doi=10.1.1.854.7516&rep=rep1&type=pdf

[4] UNICEF, AMREF, Federal Federal democratic republic of Ethiopia, Ministry of Health. Family Planning; Blended Learning Module for the Health Extension Program. Architectural Digest. (2012). p. 109–110. Available online at: https://www.open.edu/openlearncreate/mod/oucontent/view.php?id=133 (accessed December 15, 2021).

[5] Family Planning Division, Ministry of Health and Family Welfare; Government of India. Post-Abortion Family Planning Technical Update. (2016). Available online at: http://nhm.gov.in/images/pdf/programmes/familyplaning/guidelines/Post_Abortion_Family_Planning.pdf (accessed December 15, 2021).

[6] McLaurin KE Senanayake P Toubia N Ladipo OA. Post-abortion family planning. World Health Forum. (1995)16:52–5. 7873025

[7] Trends in Maternal Mortality 2000 to 2017. Estimates by WHO, UNICEF, UNFPA, Worls Bank Group, and the United Nations Population Division. Vol. 6. (2019). Available online at: https://www.unfpa.org/featured-publication/trends-maternal-mortality-2000-2017

[8] World Health Organization (WHO). Maternal Mortality Key Facts. (2019). p. 21–2. Available online at: https://www.who.int/news-room/fact-sheets/detail/maternal-mortality (accessed December 15, 2021).

[9] Ceylan A Ertem M Saka G Akdeniz N. Post abortion family planning counseling as a tool to increase contraception use. BMC Public Health. (2009) 9:1–7. 10.1186/1471-2458-9-20 19146657 PMC2651169

[10] Tessema GA Laurence CO Melaku YA Misganaw A Woldie SA Hiruye A . Trends and causes of maternal mortality in Ethiopia during 1990-2013: findings from the Global Burden of Diseases study 2013. BMC Public Health. (2017) 17:1–8. 10.1186/s12889-017-4071-8 28152987 PMC5290608

[11] World Health Organization (WHO). Unsafe Abortion, Global and Regional Estimates of the Incidence of Unsafe Abortion and Associated Mortality in 2003. Geneva: WHO (2007). p. 23–4.

[12] Ethiopia Demographic and Health Survey (EDHS). (2016). Available online at: https://dhsprogram.com/publications/publication-fr328-dhs-final-reports.cfm (accessed December 15, 2021).

[13] Induced Abortion and POSTABORTION CARE in Ethiopia. Fact sheet (2017). p. 1–5. Available online at: https://www.guttmacher.org/sites/default/files/factsheet/ethiopia_fact_sheet_final.pdf (accessed December 15, 2021).

[14] World Health Organization (WHO). Medical Eligibility Criteria for Contraceptive Use. (2010). Available online at: https://books.google.com.et/books?hl=en&lr=&id=pouTfH33wF8C&oi=fnd&pg=PP2&ots=8Y_RDZTixQ&sig=qio4S6bx9hWCGuqz_-AQBttweLM&redir_esc=y#v=onepage&q&f=false (accessed April 6, 2022).

[15] Haddad LB Nour NM. Unsafe abortion: unnecessary maternal mortality. Rev Obstet Gynecol. (2009) 2:122–6. 19609407 PMC2709326

[16] Fact Sheet. Unintended pregnancy and abortion in Uganda. Issues Brief (Alan Guttmacher Inst). (2013). 2: 1–8. 23550324

[17] Carolyn Curtis Douglas Huber TM-K. Postabortion family planning: addressing the cycle of repeat unintended pregnancy and abortion. Int Perspect Sex Reprod Health. (2010) 36:1–5. 10.1363/3604410 20403805

[18] United Nation. World Mortality Report 2005, Department of Economic and Social Affairs Population Division. ReVision (2006). Available online at: https://www.un.org/en/development/desa/population/publications/pdf/mortality/world-mortality-2005.pdf (accessed December 15, 2021).

[19] World Health Organization (WHO). Post-Abortion Family Planning; a Practical Guide for Program Managers. Geneva: WHO (2018). p. 1–8.

[20] Moges Y Hailu T Dimtsu B Yohannes Z Kelkay B. Factors associated with uptake of post-abortion family planning in Shire town, Tigray, Ethiopia. BMC Res Notes. (2018) 11:1–6. 10.1186/s13104-018-4029-7 30591074 PMC6307259

[21] Hagos G Tura G Kahsay G Haile K Grum T Araya T. Family planning utilization and factors associated among women receiving abortion services in health facilities of central zone towns of Tigray, Northern Ethiopia: a cross-sectional study. BMC Women's Health. (2018) 18:1–8. 10.1186/s12905-018-0582-4 29871631 PMC5989472

[22] Mekuria A Gutema H Wondiye H Abera M. Postabortion contraceptive use in Bahir Dar, Ethiopia: a cross-sectional study. Contracept Reprod Med. (2019) 4:1–6. 10.1186/s40834-019-0099-8 31700669 PMC6827170

[23] Muche A Bewket B Ayalew E Demeke E. Utilization of post abortal contraceptive use and associated factors among women who came for abortion service at Debre Berhan Hospital, Debre Berhan, Ethiopia March 2019: an institution-based cross-sectional study. Clin J Obstet Gynecol. (2019) 2:025–33. 10.29328/journal.cjog.1001020

[24] Kokeb L Endeshaw A Hiwot Kassa TS. Utilization of post-abortion contraceptive and associated factors among women who came for abortion service: a hospital-based cross-sectional study. J Fam Med Dis Prev. (2015) 1:4–7. 10.23937/2469-5793/1510022

[25] Seid A Gebremariam A Abera M. Integration of family planning services within post-abortion care at health facilities in Dessie –North-East Ethiopia. Sci Technol Arts Res J. (2013) 1:38. 10.4314/star.v1i1.98771

[26] Abamecha A Alemayehu Shiferaw AK. Assessment of post-abortion contraceptive intention and associated factors among abortion clients in Gambella health facilities. Val Int Journals. (2016) 3:215–25. 10.18535/ijmsci/v3i8.7

[27] Erko EK Abera M Admassu B. Safe abortion care, utilization of post-abortion contraception and associated factors, Jimma Ethiopia. J Women's Heal Care. (2016) 5:2–12. 10.4172/2167-0420.1000321

[28] Asrat M Bekele D Rominski SD. Post-abortion contraceptive acceptance and choice determinants among women receiving abortion care At Saint Paul's Hospital, Addis Ababa, Ethiopia. Ethiop J Reprod Heal. (2018) 10:35–48. 10.1016/S2214-109X(18)30166-9

[29] Prata N Holston M Fraser A Melkamu Y. Contraceptive use among women seeking repeat abortion in Addis Ababa, Ethiopia. Afr J Reprod Health. (2013) 17:56–65. 24558782

[30] Prata N Bell S Holston M Gerdts C Melkamu Y. Factors associated with choice of post-abortion contraception in Addis Ababa, Ethiopia. Afr J Reprod Health. (2011) 15:51–7. 22574492

[31] Kumbi S Melkamu Y Yeneneh H. Quality of post-abortion care in public health facilities in Ethiopia. Ethiop J Heal Dev. (2008) 22:1–8. 10.4314/ejhd.v22i1.10059

[32] Abate E Smith YR Kindie W Girma A Girma Y. Prevalence and determinants of post-abortion family planning utilization in a tertiary hospital of Northwest Ethiopia: a cross-sectional study. Contracept Reprod Med. (2020) 5:1–6. 10.1186/s40834-020-00143-4 33317644 PMC7737378

[33] Tesfaye G Oljira L. Post abortion care quality status in health facilities of Guraghe zone, Ethiopia. Reprod Health. (2013) 10:1–7. 10.1186/1742-4755-10-35 23875945 PMC3726452

[34] Wado YD Dijkerman S Fetters T. An examination of the characteristics and contraceptive acceptance of post-abortion clients in Ethiopia. Women Heal. (2021) 61:133–47. 10.1080/03630242.2020.1844358 33190621

[35] Teshome A Wondafrash M Gashawbeza B Nigatu B Asrat M Compton SD. Post-abortion contraceptive adoption in Ethiopia. Int J Gynecol Obstet. (2020) 1–5. 10.1002/ijgo.13555 33341952

[36] Muchie A Getahun FA Bekele YA Samual T Shibabaw T. Magnitudes of post-abortion family planning utilization and associated factors among women who seek abortion service in Bahir Dar Town health facilities, Northwest Ethiopia, facility-based cross-sectional study. PLoS ONE. (2021) 16:e0244808. 10.1371/journal.pone.0244808 33471864 PMC7817005

[37] Ali MM Cleland JG Shah IH World Health Organization (WHO). Causes and Consequences of Contraceptive Discontinuation : Evidence From 60 Demographic and Health Surveys. Geneva: World Health Organization (2012).

[38] Calhoun LM Speizer IS Rimal R Sripad P Chatterjee N Achyut P . Provider imposed restrictions to clients' access to family planning in urban Uttar Pradesh, India: a mixed-methods study. BMC Health Serv Res. (2013) 13:532. 10.1186/1472-6963-13-532 24365015 PMC3879325

[39] Hardee K Harris S Rodriguez M Kumar J Bakamjian L Newman K . Achieving the goal of the London summit on family planning by adhering to voluntary, rights-based family planning: what can we learn from past experiences with coercion? Int Perspect Sex Reprod Health. (2014) 40:206–14. 10.1363/4020614 25565348

[40] Schrumpf LA Stephens MJ Nsarko NE Akosah E Baumgartner JN Ohemeng-Dapaah S . Side effect concerns and their impact on women's uptake of modern family planning methods in rural Ghana: a mixed-methods study. BMC Womens Health. (2020) 20:1–8. 10.1186/s12905-020-0885-0 32192473 PMC7082910

[41] Liberati A Altman DG Tetzlaff J Mulrow C Gøtzsche PC Ioannidis JPA . The PRISMA Statement for reporting systematic reviews and meta-analyses of studies that evaluate health care interventions. PLoS Med. (2009) 6:100. 10.1371/journal.pmed.1000100 19621070 PMC2707010

[42] Moola S Munn Z Tufanaru C Aromataris E Sears K Sfetcu R . The Joanna Briggs Institute Critical Appraisal Tools for Use in JBI Systematic Reviews, Checklist for Analytical Cross-Sectional Studies. (2017). p. 1–7. Available online at: http://joannabriggs.org/research/critical-appraisal-tools.html (accessed December 15, 2021).

[43] Rücker G Schwarzer G Carpenter JR. Schumacher M. Undue reliance on I (2) in assessing heterogeneity may mislead. BMC Med Res Methodol. (2008) 9:1–9. 10.1186/1471-2288-8-79 19036172 PMC2648991

[44] Borenstein M Hedges LV Higgins JPT Rothstein HR. A basic introduction to fixed-effect and random-effects models for meta-analysis. Res Synth Methods. (2010) 1:197–111. 10.1002/jrsm.12 26061376

[45] Constitution of the Federal Democratic Republic of Ethiopia. Proclamation No. 1/1995. Fed Negarit Gaz. (1995). 1 p. Available online at: http://www.wipo.int/edocs/lexdocs/laws/en/et/et007en.pdf (accessed December 15, 2021).

[46] Kaja J Suzy Sacher SM. New security beat family planning can mean big progress for the sustainable development goals-and here's how blog Wilson Center's. Environ Change Secure Progr. (2018) 1–5. Available online at: https://www.newsecuritybeat.org/2018/07/family-planning-big-progresssustainable-development-goals-and-heres/

[47] Makenzius M Faxelid E Gemzell-Danielsson K Odero TMA Klingberg-Allvin M Oguttu M. Contraceptive uptake in post-abortion care - secondary outcomes from a randomized controlled trial, Kisumu, Kenya. PLoS ONE. (2018) 13:1–13. 10.1371/journal.pone.0201214 30096148 PMC6086397

[48] Baynes C Kahwa J Lusiola G Mwanga F Bantambya J Ngosso L . What contraception do women use after experiencing complications from abortion? An analysis of cohort records of 18,688 postabortion care clients in Tanzania. BMC Women's Health. (2019) 19:1–12. 10.1186/s12905-018-0687-9 30691443 PMC6350325

[49] Rasch V Massawe S Yambesi F Bergstrom S. Acceptance of contraceptives among women who had an unsafe abortion in Dar es Salaam. Trop Med Int Heal. (2004) 9:399–405. 10.1111/j.1365-3156.2004.01197.x 14996370

[50] Chukwumalu K Gallagher MC Baunach S Cannon A. Uptake of postabortion care services and acceptance of postabortion contraception in Puntland, Somalia. Reprod Health Matters. (2017) 25:48–57. 10.1080/09688080.2017.1402670 29231790

[51] Mina Millimouno T Delamou A Sidibé S Kolie D Pierre Leno J Delvaux T . The uptake of modern contraceptive methods among clients of post-abortion care services in Urban Guinea. Cent African J Public Heal. (2019) 5:203. 10.11648/j.cajph.20190505.14

[52] Benson J Andersen K Brahmi D Healy J Mark A Ajode A . What contraception do women use after abortion? An analysis of 319,385 cases from eight countries. Glob Public Health. (2018) 13:35–50. 10.1080/17441692.2016.1174280 27193827

[53] Benson J Andersen K Healy J Brahmi D. What factors contribute to postabortion contraceptive uptake by young women? A program evaluation in 10 countries in Asia and Sub-Saharan Africa. Glob Heal Sci Pract. (2017) 5:644–57. 10.9745/GHSP-D-17-00085 29284699 PMC5752610

[54] Azmat SK Hameed W Ishaque M Mustafa G Ahmed A. Post-abortion care family planning use in Pakistan. Pakistan J Public Health. (2012) 2:4–9. Available online at: https://ecommons.aku.edu/cgi/viewcontent.cgi?article=1896&context=pakistan_fhs_mc_chs_chs

[55] Ferreira AL Souza AI Lima RA Braga C. Choices on contraceptive methods in post-abortion family planning clinic in northeast Brazil. Reprod Health. (2010) 7:5–9. 10.1186/1742-4755-7-5 20459754 PMC2883537

[56] Banerjee SK Gulati S Andersen KL Acre V Warvadekar J Navin D. Associations between abortion services and acceptance of postabortion contraception in Six Indian States. Stud Fam Plann. (2015) 46:387–403. 10.1111/j.1728-4465.2015.00039.x 26643489 PMC5064648

[57] Shrestha A Sharma P. Post abortion choice and acceptance of contraception. Nepal J Obstet Gynaecol. (2013) 8:14–7. 10.3126/njog.v8i1.8854

[58] Federal Ministry of Health of Ethiopia. Health Sector Strategic Plan (HSDP-III) 2005/6-2009/10. Gov Ethiop. (2005). Available online at: https://extranet.who.int/countryplanningcycles/sites/default/files/planning_cycle_repository/ethiopia/ethiopia-health-sector-development-planhsdp-iii.pdf (accessed December 15, 2021).

[59] The Federal Democratic Republic of Ethiopia Ministry of Health. Health Sector Transformation Plan 2015/16 - 2019/20. (2015). Available online at: https://www.globalfinancingfacility.org/sites/gff_new/files/Ethiopia-health-system-transformation-plan.pdf (accessed December 15, 2021).

[60] López-Cevallos DF Chi C. Assessing the context of health care utilization in Ecuador:a spatial and multilevel analysis. BMC Health Serv Res. (2010) 25:209–18. 10.1186/1472-6963-10-64 20222988 PMC2850335

[61] Sjoquist P. Tanzania Demographic and Health Survey. Tanzania: Natl Bur Stat Dar es Salaam (2010). p. 163–99.

[62] Federal Democratic Republic of Ethiopia Ministry of Health. National Guideline for Family Planning Services in Ethiopia. 3rd ed. (2020). Available online at: http://repository.iifphc.org/bitstream/handle/123456789/1032/National%20Guideline%20for%20Family%20Planning%202020.pdf?sequence=1&isAllowed=y (accessed December 15, 2021).

[63] Higgins JP Altman DG. Assessing Risk of Bias in Included Studies; Cochrane handbook for Systematic Reviews of Interventions. Southern Gate: Cochrane Book Series (2008). p. 187–241.

[64] Oswald FL. Book review: publication bias in meta-analysis: prevention, assessment, and adjustments. Appl Psychol Meas. (2009) 33:74–6. 10.1177/0146621608327804

[65] Lane DM Dunlap WP. Estimating effect size: bias resulting from the significance criterion in editorial decisions. Br J Math Stat Psychol. (1978) 31:107–12. 10.1111/j.2044-8317.1978.tb00578.x

[66] Nuijten MB Van Assen MALM Veldkamp CLS Wicherts JM. The replication paradox: combining studies can decrease the accuracy of effect size estimates. Rev Gen Psychol. (2015) 19:172–82. 10.1037/gpr0000034

[67] Hip SD. High impact practices in family planning.post-abortion family planning: strengthening the family planning component of postabortion care. Fam Plan High Impact Pract. (2012) 16:52–5. Available online at: https://www.fphighimpactpractices.org/wp-content/uploads/2020/03/Postabortion-Family-Planning-EN.pdf

[68] Opoku B. Contraceptive preferences of post-abortion patients in Ghana. J Women's Heal Care. (2012) 01. 10.4172/2167-0420.1000109

[69] Carneiro Gomes Ferreira AL Impieri Souza A Evangelista Pessoa R. Braga C. The effectiveness of contraceptive counseling for women in the postabortion period: an intervention study. Contraception. (2011) 84:377–83. 10.1016/j.contraception.2011.02.003 21920193

[70] Borges ALV Olaolorun F Fujimori E Hoga LAK Tsui AO. Contraceptive use following spontaneous and induced abortion and its association with family planning services in primary health care: results from a Brazilian longitudinal study. Reprod Health. (2015) 12:4–13. 10.1186/s12978-015-0087-7 26470703 PMC4606494

[71] USAID. Counseling for Effective Use of Family Planning Trainer's Manual. (2008). 14 p. Available online at: https://www.engenderhealth.org/files/pubs/acquire-digital~archive/10.0_training_curricula_and_materials/10.2_resources/fp_curric_ph_main_text.pdf (accessed December 15, 2021).

[72] Weisman CS Maccannon DS Henderson JT Shortridge E Orso CL. Contraceptive counseling in managed care: preventing unintended pregnancy in adults. Womens Heal Issues. (2002) 12:79–95. 10.1016/S1049-3867(01)00147-5 11879761

[73] Belay AD Mengesha ZB Woldegebriel MK Gelaw YA. Married women's decision-making power on family planning use and associated factors in Mizan-Aman, South Ethiopia: a cross-sectional study. BMC Womens Health. (2016) 16:1–6. 10.1186/s12905-016-0290-x 26952021 PMC4782567

[74] Binyam B Mekitie W Tizta T Eshetu G. Married women's decision-making power on modern contraceptive use in urban and rural southern Ethiopia. BMC Public Health. (2011) 11:5–11. 10.1186/1471-2458-11-342 21595897 PMC3114727

[75] Baynes C Yegon E Lusiola G Achola J Kahando R. Post-abortion fertility desires, contraceptive uptake and unmet need for family planning: voices of post-abortion care clients in Tanzania. J Biosoc Sci. (2020). 11:342. 10.1017/S0021932020000607 33050954

[76] Lasong J Zhang Y Gebremedhin SA Opoku S Abaidoo CS Mkandawire T . Determinants of modern contraceptive use among married women of reproductive age: a cross-sectional study in rural Zambia. BMJ Open. (2020) 10:1–10. 10.1136/bmjopen-2019-030980 32234737 PMC7170561

[77] Ochako R Mbondo M Aloo S Kaimenyi S Thompson R Temmerman M . Barriers to modern contraceptive methods uptake among young women in Kenya:a qualitative study Global Health. BMC Public Health. (2015) 15:1–9. 10.1186/s12889-015-1483-1 25884675 PMC4336491

[78] Endriyas M Eshete A Mekonnen E Misganaw T Shiferaw M. Where we should focus? Myths and misconceptions of long-acting contraceptives in southern nations, nationalities and People's region, Ethiopia: qualitative study. BMC Pregn Childbirth. (2018) 18:1–6. 10.1186/s12884-018-1731-3 29653581 PMC5899320

[79] Belda SS Haile MT Melku AT Tololu AK. Modern contraceptive utilization and associated factors among married pastoralist women in Bale eco-region, Bale Zone, southeast Ethiopia. BMC Health Serv Res. (2017) 17:1–12. 10.1186/s12913-017-2115-5 28288616 PMC5348813

