# Supplementary material for: A Systematic Review and Meta-Analysis on Post-Abortion Contraceptive Utilization and Associated Factors in Ethiopia
Source: Front Public Health. 2022 May 20;10:883710. doi: 10.3389/fpubh.2022.883710 (PMC9163437; doi:10.3389/fpubh.2022.883710)
Supplement: Supplementary file 1 [file Table_1.DOCX]

Supplementary file 1. Quality score of included studies in this review to estimate the pooled prevalence of post-abortion contraceptive utilization and its associated factors in Ethiopia.

|  |  | Assessment criteria's for quality of the primary studies | | | | | | | | | |  | |  | |
| --- | --- | --- | --- | --- | --- | --- | --- | --- | --- | --- | --- | --- | --- | --- | --- |
| s/no | studies | (1): Where the criteria for inclusion in the sample clearly defined?  1=Yes  0= No | (2): Were the study subjects and the setting described in detail?  1=Yes  0= No | (3): Was the exposure measured validly and reliably?  1=Yes  0= No | (4): Were objective, standard criteria used for measurement of the condition?  1=Yes  0= No | (5): Were confounding factors identified?  1=Yes  0= No | (6): Were strategies to deal with confounding factors stated?  1=Yes  0= No | (7): Were the outcomes measured in a valid and reliable way?  1=Yes  0= No | (8): Was an appropriate statistical analysis used?  1=Yes  0= No | Total grade in number(summation of 1-8) | 100% | | Rank | |  |
| 1 | Moges Y et al[20] | 1 | 1 | 1 | 1 | 0 | 1 | 1 | 1 | 7 | 87.5 | | high | |  |
| 2 | hagos G et al[21] | 1 | 1 | 0 | 0 | 1 | 1 | 1 | 1 | 6 | 75 | | medium | |  |
| 3 | Mekuria A et al[22] | 1 | 1 | 1 | 1 | 0 | 1 | 1 | 1 | 7 | 87.5 | | high | |  |
| 4 | Muche A et al[23] | 1 | 1 | 1 | 1 | 1 | 1 | 1 | 1 | 8 | 100 | | high | |  |
| 5 | Kokeb L et al[24] | 0 | 1 | 1 | 1 | 0 | 1 | 1 | 1 | 6 | 75 | | medium | |  |
| 6 | Awol S et al[25] | 1 | 1 | 1 | 1 | 1 | 1 | 1 | 1 | 8 | 100 | | high | |  |
| 7 | Abamecha A et al[26] | 1 | 1 | 0 | 0 | 0 | 1 | 1 | 1 | 5 | 62.5 | | medium | |  |
| 8 | Erko EK et al[27] | 1 | 1 | 0 | 0 | 1 | 1 | 1 | 1 | 6 | 75 | | medium | |  |
| 9 | Asrat M et al[28] | 1 | 1 | 0 | 0 | 0 | 1 | 1 | 1 | 5 | 62.5 | | medium | |  |
| 10 | Prata N et al[29] | 1 | 1 | 1 | 1 | 0 | 1 | 1 | 1 | 7 | 87.5 | | high | |  |
| 11 | Prata N et al[30] | 0 | 1 | 1 | 1 | 1 | 1 | 1 | 1 | 7 | 87.5 | | high | |  |
| 12 | Kumbi S et al[31] | 0 | 1 | 1 | 1 | 0 | 0 | 1 | 1 | 5 | 62.5 | | medium | |  |
| 13 | Abate E et al[32] | 1 | 1 | 1 | 1 | 1 | 1 | 1 | 1 | 8 | 100 | | high | |  |
| 14 | Tesfaye G et al[33] | 1 | 1 | 1 | 1 | 0 | 0 | 1 | 1 | 6 | 75 | | medium | |  |
| 15 | Wado Y et al[34] | 1 | 0 | 1 | 1 | 1 | 1 | 1 | 1 | 7 | 85.5 | | high | |  |
| 16 | Teshome A et al[35] | 1 | 1 | 1 | 1 | 0 | 1 | 1 | 1 | 7 | 85.5 | | high | |  |
| 17 | Muchie A et al[36] | 1 | 1 | 1 | 1 | 1 | 1 | 1 | 1 | 8 | 100 | | high | |  |
| We included all primary studies with a quality score of ≥50% in the analysis  Overall appraisal: 17 original studies  Included: 17 original studies with a quality score of ≥50% | | | | | | | | | | | | | |  | |
